# Removing Tannins from Medicinal Plant Extracts Using an Alkaline Ethanol Precipitation Process: A Case Study of Danshen Injection

**DOI:** 10.3390/molecules191118705

**Published:** 2014-11-14

**Authors:** Xingchu Gong, Yao Li, Haibin Qu

**Affiliations:** Pharmaceutical Informatics Institute, College of Pharmaceutical Sciences, Zhejiang University, Hangzhou 310058, China

**Keywords:** alkaline ethanol precipitation, tannin removal, phenolic compound, mechanism, model

## Abstract

The alkaline ethanol precipitation process is investigated as an example of a technique for the removal of tannins extracted from *Salviae miltiorrhizae Radix et Rhizoma* for the manufacture of Danshen injection. More than 90% of the tannins can be removed. However, the recoveries of danshensu, rosmarinic acid, and salvianolic acid B were less than 60%. Total tannin removal increased as the refrigeration temperature decreased or the amount of NaOH solution added increased. Phenolic compound recoveries increased as refrigeration temperature increased or the amount of NaOH solution added decreased. When operated at a low refrigeration temperature, a relative high separation selectivity can be realized. Phenolic compound losses and tannin removal were mainly caused by precipitation. The formation of phenol salts, whose solubility is small in the mixture of ethanol and water used, is probably the reason for the precipitation. A model considering dissociation equilibrium and dissolution equilibrium was established. Satisfactory correlation results were obtained for phenolic compound recoveries and total tannin removal. Two important parameters in the model, which are the water content and pH value of alkaline supernatant, are suggested to be monitored and controlled to obtain high batch-to-batch consistency.

## 1. Introduction

Tannins, polyphenolic compounds with relative large molecular weight, widely exist in medicinal plants and are considered to have antioxidant properties. However, the presence of tannins in botanical extract injections may cause the swelling and redness at the injection site, cardiac arrhythmia, and anaphylactic shock [1,2,3], therefore they are considered as toxic impurities in botanical injections [4], making the removal of tannins matter of significant concern in the production of botanical injections. 

Tannins can be removed by alkaline ethanol precipitation, electrochemical oxidation [5,6], catabolic enzyme reaction [7], electroprecipitation [8], or adsorption [4,9,10,11,12]. Alkaline ethanol precipitation is a traditional method used in the manufacture of botanical injections, such as Danshen injection and Guanxinning injection. It can be realized by simply adding NaOH solution to the supernatant obtained using an ethanol precipitation process. The operation is easy and the solvent is safe. Comparison of tannin contents of Guanxinning injections manufactured using and without using alkaline ethanol precipitation process shows that the former is much lower than the latter [13]. Recently, the ethanol precipitation process dealing with botanical extracts has gained much attention because of its wide usage in the traditional Chinese medicine and food industries [14,15,16,17,18,19], but research on the alkaline ethanol precipitation process is very limited. The mechanism of tannin removal has not been verified and the effects of different factors on the active constituents in botanical extracts are not clear. 

**Figure 1:**
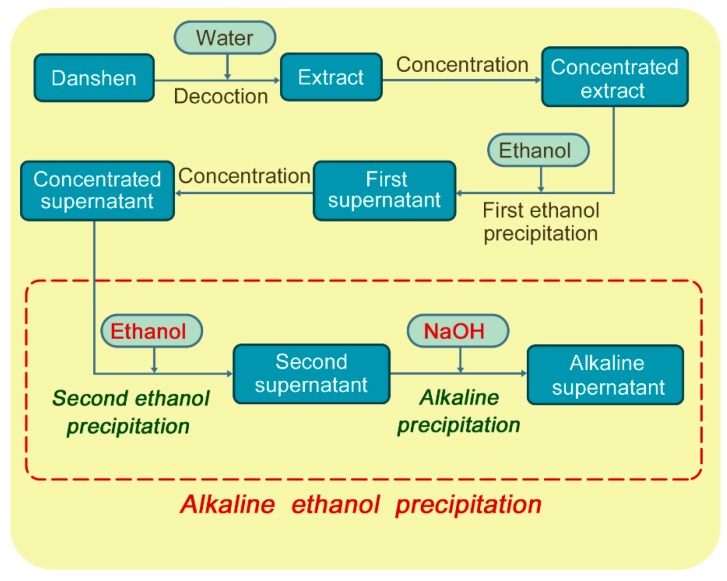
Schematic diagram of the alkaline ethanol precipitation process.

The investigation on the alkaline ethanol precipitation process can improve pharmaceutical process understanding, and further help implement the concept of Quality by Design (QbD) to improve drug quality control [20]. Therefore, the alkaline ethanol precipitation process in the manufacture of Danshen injection, as seen in Figure 1, was investigated as an example in this work. 

**Figure 2:**
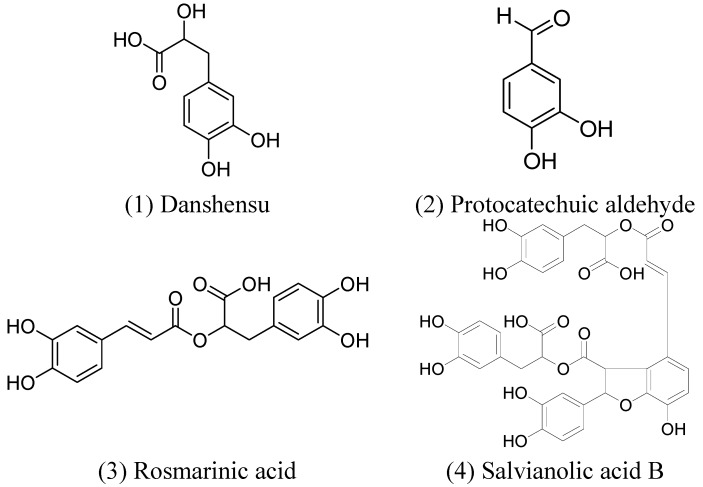
Structural formulae of some active components of Daishen [27].

Danshen injection is a botanical injection made from *Salviae miltiorrhizae Radix et Rhizoma* (Danshen in Chinese). It is clinically used for the treatment of coronary artery disease and angina pectoris [21]. According to the pharmacology studies, phenolic compounds such as danshensu (DSS), protocatechuic aldehyde (PA), rosmarinic acid (RA), and salvianolic acid B (SaB) are considered the active constituents of Danshen injection [22,23,24,25,26]. Their structures are shown in Figure 2. 

In this work, the effects of four factors of the alkaline ethanol precipitation process, including refrigeration temperature, the amount of ethanol added, the amount of alkali added, and refrigeration time were investigated. Phenolic compound contents and total tannin content in the alkaline supernatants were determined. The recoveries of phenolic compounds and the removal of total tannin were calculated. The mechanisms of phenolic compound loss and tannin removal were discussed. A mathematical model based on dissociation equilibrium and dissolution equilibrium was established to correlate the experimental data.

## 2. Results and Discussion

### 2.1. Composition of the Concentrated Supernatant

Concentrated supernatant is the material of the alkaline ethanol precipitation process, as seen in Figure 1. The contents of phenolic compounds, total tannin, dry matter, and water content in the concentrated supernatant are listed in Table 1. Dry matter in the concentrated supernatant was mainly composed of phenolic compounds and saccharides [17]. DSS content was higher than any other phenolic compound content in the concentrated supernatant. The sum of four phenolic compounds was 59.5 mg/g, which was 10.0% of dry matter. The mass ratio of total active constituent and total tannin (RACTT) value of the concentrated supernatant was 10.4.

**Table 1:** Composition of the concentrated supernatant

Composition	Content (mg/g)	Composition	Content (mg/g)
Dry matter	593.7	Water content	365.1
DSS	27.2	PA	4.18
RA	5.02	SaB	23.1
Total tannin	5.73		

### 2.2. Effects of Refrigeration Time

**Figure 3:**
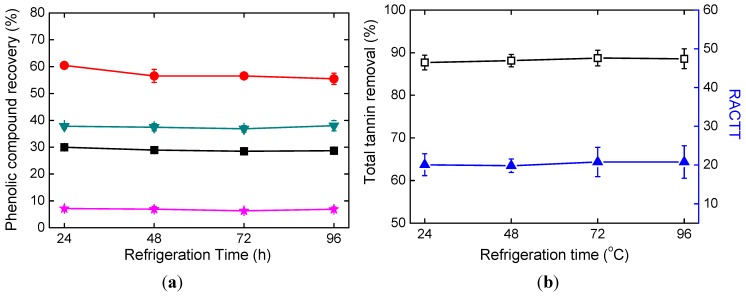
Effects of refrigeration time. (**a**) Phenolic compound recoveries; (**b**) Total tannin removal and RACTT values.

According to Figure 3a, refrigeration time showed little effects on DSS recovery, RA recovery, SaB recovery, and total tannin removal (TTR). 

*n* = 3; ■, DSS; ●, PA; ▼, RA; ★, SaB; □, Total tannin removal; ▲, RACTT.

In Table 2, the corresponding p values are higher than 0.05. The recoveries of DSS and RA were less than 40%. SaB recovery was less than 10%. It can be concluded that serious losses of active constituents were observed. TTR and RACTT values changed little as refrigeration time increased, as seen in Figure 3b. RACTT values were larger than that of the concentrated supernatant, which indicates that drug safety was improved. 

**Table 2:** P values obtained with ANOVA method.

Parameters	Refrigeration Time	Refrigeration Temperature	Ethanol Amount	NaOH Solution Amount
TTR	0.899	0.001 ^a^	0.000 ^a^	0.000 ^a^
PCRDSS	0.245	0.000 ^a^	0.000 ^a^	0.000 ^a^
PCRPA	0.030	0.000 ^a^	0.002 ^a^	0.000 ^a^
PCRRA	0.769	0.001 ^a^	0.000 ^a^	0.000 ^a^
PCRSaB	0.567	0.000 ^a^	0.000 ^a^	0.000 ^a^
RECTT	0.975	0.006 ^a^	0.156	0.003 ^a^

^a^ *p*-value less than 0.01.

### 2.3. Effects of Refrigeration Temperature 

**Figure 4:**
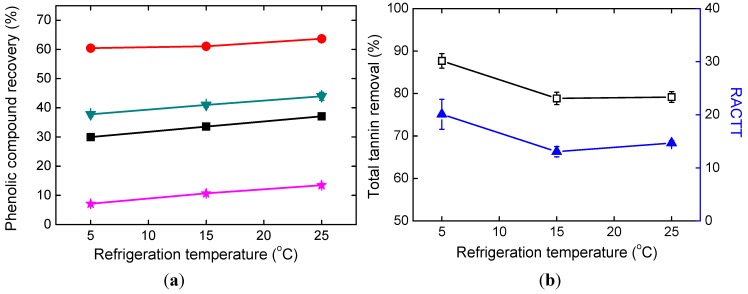
Effects of refrigeration temperature. (**a**) Phenolic compound recoveries; (**b**) Total tannin removal and RACTT values.

Refrigeration temperature significantly affected PCR and TTR with p values less than 0.01, as seen in Table 2. In Figure 4a, the recoveries of all the four phenolic compounds of DSS, PA, RA, and SaB slightly increased as refrigeration temperature increased. Higher total tannin removal and higher RACTT values were obtained when the refrigeration temperature was 5 °C, as seen in Figure 4b. Comparing with that of the concentrated supernatant, the RACTT value increased more at 5 °C. Therefore lower temperature is favored in the alkaline ethanol precipitation.

*n* = 3; ■, DSS; ●, PA; ▼, RA; ★, SaB; □, Total tannin removal; ▲, RACTT.

### 2.4. Effects of Alkali Addition

**Figure 5:**
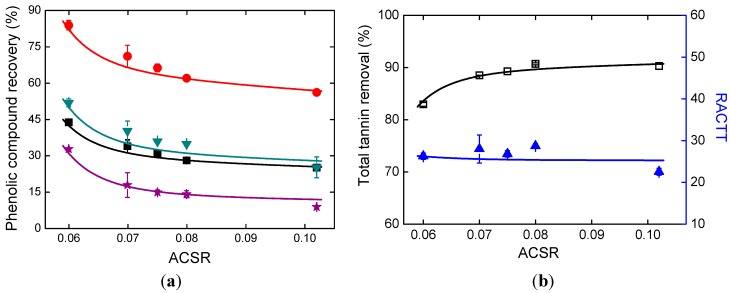
Effects of alkali addition. (**a**) Phenolic compound recoveries; (**b**) Total tannin removal and RACTT values.

The amount of NaOH solution added is also a significant factor for the alkaline ethanol precipitation process. All the p values are less than 0.01 in Table 2. In Figure 5a, ACSR refers to the mass ratio of NaOH solution and the concentrated supernatant. The recoveries of all the four phenolic compounds decreased as the amount of NaOH solution added increased. PA recovery was higher than that of any other phenolic acids. SaB, which is a dicarboxylic acid, showed the lowest recovery. Though more phenolic compounds were lost when the amount of NaOH solution added increased, RACTT values were higher than 20, as shown in Figure 5b. This should be attributed to the increasing removal of tannins. In Figure 5b, the tannin removal values were more than 80% and increased remarkably as the amount of NaOH solution added increased. 

*n* = 3; ■, DSS; ●, PA; ▼, RA; ★, SaB; □, Total tannin removal; ▲, RACTT; Solid lines, calculation results.

### 2.5. Effects of Ethanol Addition

**Figure 6:**
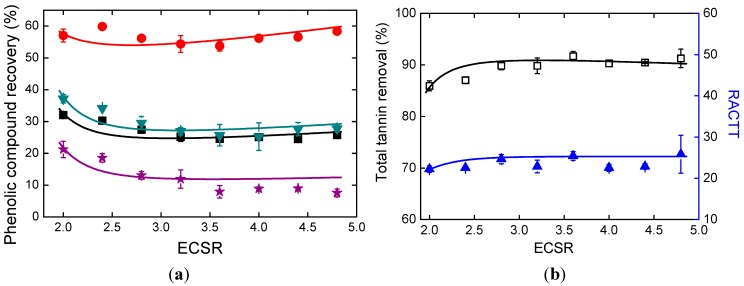
Effects of ethanol addition. (**a**) Phenolic compound recoveries; (**b**) Total tannin removal and RACTT values.

The amount of ethanol added is considered to be another important factor because it affects the solvent composition of the supernatant. In Table 2, all the p values are less than 0.01. In Figure 6a, ECSR represents the mass ratio of ethanol and the concentrated supernatant. PA recovery, which was higher than 50%, was the highest among all the phenolic compound recoveries. SaB recovery was lower than 22%. RA recovery and SaB recovery decreased slightly as ethanol addition increased. Figure 6b shows the effects of ethanol addition on TTR and RACTT values. RACTT values varied little. TTR was between 85% and 92%, and slightly increased when ECSR value was less than 3. 

*n* = 3; ■, DSS; ●, PA; ▼, RA; ★, SaB; □, Total tannin removal; ▲, RACTT; Solid lines, calculation results.

### 2.6. Mechanism of Phenolic Compound Loss and Tannin Removal

Phenolic compounds may be lost because of chemical transformation or precipitation during the alkaline ethanol precipitation process [28]. To examine this hypothesis the precipitate of Experiment 13 in Table 3 was analyzed. 

**Table 3:** Experimental conditions, supernatant pH value, and MSPCS values (*n* = 3).

NO.	ECSR	Refrigeration Temperature (°C)	Refrigeration Time (h)	ACSR	Alkaline Supernatant pH Value	MSPCS (g/g)
1	2.0	5	24	0.102	8.614 ± 0.614	2.418 ± 0.001
2	2.4	5	24	0.102	8.658 ± 0.031	2.753 ± 0.005
3	2.8	5	24	0.102	8.937 ± 0.044	3.026 ± 0.003
4	3.2	5	24	0.102	9.024 ± 0.175	3.257 ± 0.062
5	3.6	5	24	0.102	9.185 ± 0.119	3.511 ± 0.045
6	4.0	5	24	0.102	9.101 ± 0.067	3.827 ± 0.018
7	4.4	5	24	0.102	9.046 ± 0.041	4.054 ± 0.052
8	4.8	5	24	0.102	9.150 ± 0.089	4.482 ± 0.082
9	4.0	5	24	0.060	7.350 ± 0.022	4.416 ± 0.016
10	4.0	5	24	0.070	8.036 ± 0.134	4.289 ± 0.053
11	4.0	5	24	0.075	8.282 ± 0.115	4.227 ± 0.026
12	4.0	5	24	0.080	8.413 ± 0.097	4.167 ± 0.034
13	4.0	5	24	0.082	8.483 ± 0.042	4.170 ± 0.005
14	4.0	5	48	0.082	8.532 ± 0.070	4.156 ± 0.089
15	4.0	5	72	0.082	8.413 ± 0.047	4.248 ± 0.028
16	4.0	5	96	0.082	8.412 ± 0.096	4.214 ± 0.058
17	4.0	15	24	0.082	8.550 ± 0.047	4.293 ± 0.013
18	4.0	25	24	0.082	8.369 ± 0.014	4.425 ± 0.020

**Scheme 1:**
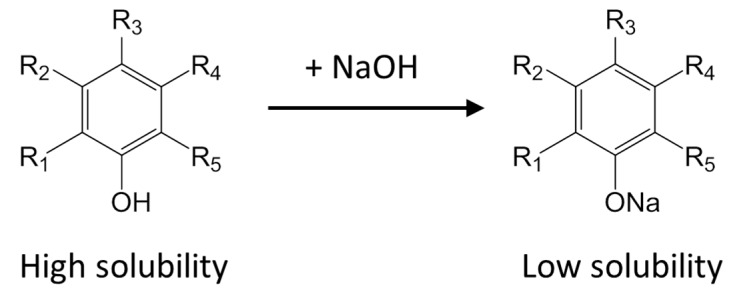
The mechanism of phenolic compound loss and tannin removal.

All four phenolic compounds were found in the precipitate. Considering that the increase of NaOH solution addition amount resulted in lower phenolic compound recoveries, phenolic compounds probably formed sodium salts. The solubility of these salts are small in the mixture of ethanol and water, which results in precipitation. The mechanism is shown in Scheme 1. 

The precipitated phenolic compound (RPPC) ratio represents the proportion of phenolic compounds which is lost because of precipitation, as seen in Table 4. The RPPC value of SaB was higher than 90%, indicating most of the SaB loss was caused by precipitation. The sum of phenolic compound recovery (PCR) and RPPC is the quotient of the total amount of a phenolic compound after and before the alkaline ethanol precipitation process. To estimate the influences of chemical transformation on amounts of phenolic compounds, the sum of PCR and RPPC was calculated and listed in Table 4. For DSS, RA, and SaB, the sum of PCR and RPPC was close to 1, which means that chemical transformation had little influence on the amounts of these phenolic compounds. The sum of PCR and RPPC was about 90% for PA, which means that some of PA lost because of reactions. In Figure 3a, PA recovery decreased slightly as refrigeration time increased.

**Table 4:** RPPC and the sum of PCR and RPPC

Phenolic Compounds	RPPC (%)	PCR + RPPC (%)
DSS	67.3 ± 6.5	97.2 ± 6.2
PA	29.3 ± 3.0	89.8 ± 3.4
RA	59.7 ± 6.4	97.5 ± 6.4
SaB	91.6 ± 8.9	98.7 ± 9.1

Tannins were also found in the precipitate of Experiment 13. The ratio of precipitated tannins (PRTT) was 80.0% ± 2.1%. It indicates that most tannins formed sodium salts during the alkaline ethanol precipitation process. Total tannin remained in supernatant (TTS) can be calculated with Equation (1):

(1) TTS=1-TTR

The sum of TTS and RPTT value was 108% ± 1%. It can be concluded that the impact of chemical transformation on tannin amount was small. Because phenolic active ingredients and tannins have similar properties, the mechanisms of phenolic compound loss and tannin removal are similar. Therefore selective removal of tannins is still an unresolved technical issue.

### 2.7. Modeling 

Modeling is a way to find the potential important parameters in a process [29]. A model is derived to describe the concentrations of DSS, PA, RA, SaB, and TT in the alkaline supernatants. Precipitate generated in alkaline precipitation is assumed to contain no water. Water mass fraction in the mixture of ethanol and water of alkaline supernatant (*Ø*) then can be calculated with Equation (2) according to the law of conservation of mass.

(2) $$\phi =\frac{w_{2}+w_{1}\times \text{ECSR}+w_{3}\times \text{ACSR}}{w_{2}+w_{4}+\text{ECSR}+w_{3}\times \text{ACSR}}$$

where *w*_1_, *w*_2_, and *w*_3_ refer to the water mass fraction in 95% (v/v) ethanol, the concentrated supernatant, and NaOH solution, respectively; *w*_4_ refers to ethanol mass fraction in the concentrated supernatant. A phenolic compound exists in alkaline supernatant both in its ionized form and unionized form [28]. Therefore Equation (3) can be obtained:

(3) $${\text{PC}}_{\text{AS}}={\text{PC}}_{\text{IF}}+{\text{PC}}_{\text{UF}}$$

where PC is the phenolic compound content; subscript AS refers to the alkaline supernatant; subscripts IF and UF refer to ionized form and unionized form, respectively. There is a dissociation equilibrium between the two forms of phenolic compounds in the alkaline supernatant. By assuming that the activity coefficients of phenolic compounds were 1, Equation (4) can be obtained:

(4) $$K_{a}=\frac{{\text{A}}_{\text{IF}}\times {\text{A}}_{{\text{H}}^{\text{+}}}}{{\text{A}}_{\text{UF}}}=\frac{\gamma_{IF}}{\gamma_{UF}}\times \frac{{\text{PC}}_{\text{IF}}}{{\text{PC}}_{\text{UF}}}\times {\text{A}}_{{\text{H}}^{\text{+}}}=\frac{{\text{PC}}_{\text{IF}}}{{\text{PC}}_{\text{UF}}}\times {\text{A}}_{{\text{H}}^{\text{+}}}$$

where *K_a_* is dissociation coefficient, A is activity, and *γ* is the activity coefficient. Equation (5) can be derived from Equations (3) and (4):

(5) $${\text{PC}}_{\text{AS}}={\text{PC}}_{\text{IF}}\times K_{a}^{-1}\times {\text{A}}_{{\text{H}}^{\text{+}}}+{\text{PC}}_{\text{IF}}$$

According to Espinosa *et al.*’s work [30], *K_a_* can be assumed to be a function of *Ø*, as shown in Equation (6):

(6) $$pK_{a}=pK_{a,Eth}+\alpha \times \phi$$

where *K_a,Eth_* is the dissociation coefficient of phenolic compounds in pure ethanol and *α* is a slope parameter. Because the ionized form of a phenolic compound is considered to be saturated in the alkaline supernatant, PC_IF_ is equal to the solubility of ionized phenolic compound (*S*). *S* is assumed to increase linearly as *Ø* increases, as shown in Equation (7):

(7) $$S=S_{Eth}+\beta \times \phi$$

where *S*_Eth_ is the solubility of ionized phenolic compound in pure ethanol and *β* is a slope parameter. Accordingly, Equation (8) can be derived from Equations (5)–(7):

(8) $${\text{PC}}_{\text{AS}}=\left(S_{\text{Eth}}+\beta \times \phi \right)\left(1+\frac{{\text{A}}_{{\text{H}}^{\text{+}}}}{10^{-\left(pK_{a,Eth}+\alpha \times \phi \right)}}\right)$$

Equation (8) was applied to calibrate *K_a,Eth_*, *α*, and *β* by minimizing the average absolute deviations (AAD) values for the concentrations of four phenolic compounds and total tannin. AAD values were calculated with Equation (9):

(9) $$\text{AAD}=\frac{\sum \left|{\text{PC}}_{\text{Exp}}-{\text{PC}}_{\text{Cal}}\right|}{\text{NED}}\times 100\%$$

where NED is the number of experimental data; the subscripts Exp and Cal refer to experimental values and calculated values, respectively. In the calibration, *S*_Eth_ in Equation (8) was assumed to be zero. The calibrated results are listed in Table 5. Average relative deviations (ARD) values were also calculated with Equation (10) for comparison:

(10) $$\text{ARD}=\frac{\sum \left|\frac{{\text{PC}}_{\text{Exp}}-{\text{PC}}_{\text{Cal}}}{{\text{PC}}_{\text{Exp}}}\right|}{\text{NED}}\times 100\%$$

AAD values and ARD values are listed in Table 5.

**Table 5:** Calibrated parameters and deviations.

Phenolic Compounds	DSS	PA	RA	SaB	TT
*K_a,Eth_*	4.78	5.20	4.88	5.28	4.52
*α*	14.7	10.5	14.6	14.2	16.7
*β* (mg/g)	10.9	3.75	2.20	4.27	0.836
AAD (mg/g)	0.102	0.0198	0.0307	0.119	0.0132
ARD (%)	4.37	2.71	6.77	18.4	7.32

*K_a,Eth_* values for all the compounds are between 4 and 6. The *β* value of DSS is higher than that of other phenolic compounds, which means that DSS salt solubility in supernatant increases more rapidly as *Ø* increased. The ARD values are less than 8% for DSS, PA, RA, and TT, which means satisfactory correlation results were obtained. In this model, *Ø*, and supernatant pH value are two important parameters affecting the results of the alkaline ethanol precipitation. Therefore these two parameters should be monitored in the alkaline ethanol precipitation process to obtain a high batch-to-batch consistency. 

The mass of supernatant obtained from per gram concentrated supernatant (MSPCS) were correlated with ECSR and ACSR using Equations (11) and (12), respectively:

(11) $$\text{MSPCS}=a_{0}+a_{1}\times \text{ECSR}+a_{2}\times \text{ACSR}$$

(12) $$\text{pH}=b_{0}+b_{1}\times {\text{ECSR}}^{-1}+b_{2}\times {\text{ACSR}}^{-1}$$

where *a_0_*, *a_1_*, *a_2_*, *b_0_*, *b_1_*, and *b_2_* are calibrated parameters. The calibration results are listed in Table 6. 

**Table 6:** Calibration results for the supernatant pH value and MSPCS.

Parameters	Values	*P* value	R^2^_adj_
*a_0_*	2.456	0.000	0.996
*a_1_*	0.704	0.000	
*a_2_*	−14.04	0.000	
*b_0_*	12.05	0.000	0.980
*b_1_*	−2.02	0.000	
*b_2_*	−0.249	0.000	

The p values are less than 0.01, indicating the corresponding terms are significant. The positive value of *a_1_* means that higher amount of ethanol added leads to more supernatant. The negative value of *a_2_* means that higher amount of NaOH solution added leads to more precipitation. The values of *b_1_* and *b_2_* are negative, which means that the addition of NaOH solution and ethanol both result in supernatants with higher pH values. 

With the obtained parameters *K_a,Eth_*, *α*, *β*, *a_0_*, *a_1_*, *a_2_*, *b_0_*, *b_1_*, and *b_2_*, phenolic compound recoveries and total tannin removal can be correlated. RACTT values can also be calculated. The calculation results are shown in Figure 5 and Figure 6. The correlation results showed good agreement with the experimental results. Because of the experimental design, the prediction results of supernatant pH value and MSPCS will not be reliable when the values of ECSR and ACSR change simultaneously, therefore the optimization of parameters is not carried out in this work. 

## 3. Experimental Section 

### 3.1. Materials and Chemicals

The concentrated supernatant was kindly provided by a Chinese pharmaceutical manufacturer (Qingchunbao, Zhejiang, China). Ethanol (>99.7%) was purchased from Tianjin Damao Chemical Regent Factory (Tianjin, China). Standard substances including DSS and PA were obtained from the National Institute for the Control of Pharmaceutical and Biological Products (Beijing, China). RA and SaB (˃98%) were purchased from Winherb Medical S&T Development Co., Ltd. (Shanghai, China). Casein (AR) and Na_2_CO_3_ (AR) were purchased from Sangon Biotech Co., Ltd. (Shanghai, China). Gallic acid (AR) was obtained from Shanghai Chemical Reagent Company (Shanghai, China). HCl solution (36.0%–38.0%) was supplied by Hangzhou Chemical Reagent Co., Ltd. (Zhejiang, China). NaOH (>96.0%) was purchased from Zhongxing Chemical Reagent Co., Ltd. (Zhejiang, China). Folin & Ciocalteu’s phenol reagent was obtained from Shanghai Siji Biological Product Co., Ltd. (Shanghai, China). Formic acid (>99.0%) was purchased from the Tedia Company (Fairfield, OH, USA). Deionized water was produced using a Milli-Q academic water purification system (Millipore, Milford, MA, USA). HPLC-grade acetonitrile was purchased from Merck (Darmstadt, Germany). All materials were used as received without any further purification. 

### 3.2. Procedures

In industry, 95% (v/v) ethanol is usually used to obtain an alkaline supernatant with apparent ethanol content more than 80% (v/v). Therefore 95% (v/v) ethanol is used in this work. Ethanol was added into the concentrated supernatant in a conical flask under magnetic stirring with a flow rate of 3.6 g/min. After the addition of ethanol, a volume of 35% (m/m) NaOH solution was added into the second supernatant. After the addition of NaOH, the stirring continued for 20 min. The flasks then were refrigerated in a low-temperature thermostat bath (THD-1008W, Ningbo Tianheng Instrument Factory, Ningbo, China). The amount of ethanol added, the amount of NaOH solution added, refrigeration temperature and refrigeration time are listed in Table 3. After that, the alkaline supernatant was collected and weighed. The pH values of the alkaline supernatants were determined with a pH meter (S40, Mettler-Toledo Instruments Co., Ltd., Shanghai, China). The alkaline supernatants then were acidified with HCl solution for preservation. The concentrations of phenolic compounds and tannins were determined. The experimental conditions were designed according to industry experiences. All the experiments were repeated three times (*n* = 3).

The following equations were used to calculate MSPCS, TTR, PCR, RPPC, and RPTT:

(13) $$\text{MSPCS}=\frac{\text{MAS}}{\text{MCS}}$$

where MAS and MCS are the mass of the alkali supernatant and the mass of the concentrated supernatant, respectively:

(14) $$\text{TTR}=\left(1-\frac{{\text{TT}}_{\text{AS}}}{{\text{TT}}_{\text{CS}}}\times \text{MSPCS}\right)\times 100\%$$

where TT is the total tannin content; subscripts AS and CS refer to the alkaline supernatant and the concentrated supernatant, respectively:

(15) $$\text{PCR}=\frac{{\text{PC}}_{\text{AS}}}{{\text{PC}}_{\text{CS}}}\times \text{MSPCS}\times 100\%$$

(16) $$\text{RPPC}=\frac{{\text{PC}}_{\text{PRE}}\times \text{MPRE}}{{\text{PC}}_{\text{CS}}\times \text{MCS}}\times 100\%$$

where MPRE is the mass of precipitation, and subscript PRE represents the precipitation:

(17) $$\text{RPTT}=\frac{{\text{TT}}_{\text{PRE}}\times \text{MPRE}}{{\text{TT}}_{\text{CS}}\times \text{MCS}}\times 100\%$$

Total active constituent (TAC) is defined as the sum of DSS content, PA content, RA content, and SaB content. RACTT value in the supernatant was calculated with Equation (18):

(18) $$\text{RACTT}=\frac{\text{TAC}}{\text{TT}}$$

Higher RACTT value means smaller risks for patients when the same dosage is used. The difference of RACTT values before and after the alkaline ethanol precipitation process can represent the contribution of the ethanol precipitation process on drug safety. Analysis of Variance (ANOVA) was used to determine the impacts of refrigeration time, refrigeration temperature, ethanol amount, and NaOH solution amount on TTR, PCR, and RACTT, respectively. The results are listed in Table 2.

### 3.3. Analytical Methods

The HPLC-UV method developed in previous work [31] was used to determine the concentrations of DSS, PA, RA, and SaB. An Agilent 1100 series HPLC apparatus (Agilent Technologies, Waldbronn, Germany) was used to analyze the samples. Chromatographic separations were carried out on a Kromasil 100-5 C_18_ column (250 mm × 4.6 mm i.d., 5.0 um particle size) purchased from AkzoNobel (Stockholm, Sweden). The mobile phase consisted of solvent A (0.05% trifluoroacetic acid in water) and solvent B (acetonitrile). The gradient elution was as follows: 2.0%–30.0% B at 0–65 min; 30.0%–60.0% B at 65–75 min. The flowrate of mobile phase was 0.8 mL/min and the injection volume was 10 µL. The column temperature was set at 40 °C and detection wavelength was set at 288 nm. Total tannin content was determined using a spectrophotometric method. A mixed NaOH-Na_2_CO_3_ solution was prepared by mixing equal volume of NaOH solution (0.03 mol/L) and Na_2_CO_3_ solution (wt. 4%). Gallic acid was weighed accurately and dissolved in water to establish a standard curve. The mixed NaOH-Na_2_CO_3_ solution, gallic acid solution and Folin Ciocalteu phenol reagent were mixed and shaken for 30 min in a water bath oscillator. Spectrophotometry absorbance was measured at 760 nm. When determining the total phenol content of samples, the mixed NaOH-Na_2_CO_3_ solution, sample solution and Folin Ciocalteu phenol reagent were mixed together and measured the spectrophotometry absorbance with a similar process. Casein was applied to adsorb the tannins in samples. After adsorption, the total phenol contents of samples were also determined. The difference of total phenol contents for a sample before and after adsorption by casein was considered as the total tannin content.

## 4. Conclusions

In this work, the alkaline ethanol precipitation process used in the manufacture of Danshen injection was investigated as an example of tannin removal. More than 90% of the tannins can be removed in the alkaline ethanol precipitation process, however there was a concurrent dramatic loss of phenolic compounds. Higher refrigeration temperature and lower added amount of NaOH solution both led to lower total tannin removal and higher phenolic compound recoveries. High RACTT values were obtained when refrigeration temperature was low. The loss of phenolic compounds and the removal of tannins were both mainly caused by precipitation. A model based on dissociation equilibrium and dissolution equilibrium was established. Three parameters of *pK_a,Eth_*, *α*, and *β* were calibrated. Average relative deviations for the concentrations of PA, DSS, RA, and total tannin were less than 8%. Supernatant water content and supernatant pH value are two important parameters in the model. They are suggested to be monitored and controlled in the alkaline ethanol precipitation process. The results of this work increase the process understanding for the botanical injections manufactured using alkaline ethanol precipitation to remove tannins, which meets the requirements of QbD concept. 

## Abbreviations

A: activity
ACSR: the mass ratio of NaOH solution and the concentrated supernatant
AAD: the average absolute deviations [mg/g]
ARD: the average relative deviations [%]
DSS: Danshensu
ECSR: the mass ratio of ethanol and the concentrated supernatant
K_a_: dissociation equilibrium
MAS: mass of supernatant [g]
MCS: mass of the concentrated supernatant [g]
MPRE: the mass of precipitation [g]
MSPCS: the mass of supernatant obtained from per gram concentrated supernatant [g/g]
NED: the number of experimental data
PA: protocatechuic aldehyde
PC: phenolic compound content [mg/g]
PCR: phenolic compound recovery [%]
QbD: quality by design
RA: rosmarinic acid
RACTT: the mass ratio of total active constituent and total tannin
RPPC: the ratio of precipitated phenolic compound [%]
RPTT: the ratio of precipitated tannins [%]
S: the solubility of HIPC [mg/g]
SaB: Salvianolic acid B
TAC: total active constituent [mg/g]
TT: total tannin content [mg/g]
TTR: the total tannin removal [%]
TTS: total tannin remained in supernatant [%]
*w*_1_: water mass fraction in 95% (v/v) ethanol [%]
*w*_2_: water mass fraction in the concentrated supernatant [%]
*w*_3_: water mass fraction in NaOH solution [%]
*w*_4_: ethanol mass fraction in the concentrated supernatant [%]
*a*_0_: parameter calibrated to correlate MSPCS
*a*_1_: parameter calibrated to correlate MSPCS
*a*_2_: parameter calibrated to correlate MSPCS
*b*_0_: parameter calibrated to correlate pH
*b*_1_: parameter calibrated to correlate pH
*b*_2_: parameter calibrated to correlate pH

## Greek Symbols

*α*: slope parameter for pKa calculation
*β*: slope parameter for solubility calculation [mg/g]
*γ*: activity coefficient
*Ø*: water mass fraction in the mixture of ethanol and water of alkaline supernatant [%]

## Subscripts

AS: alkaline supernatant
Cal: calculated values
CS: the concentrated supernatant
Eth: ethanol
Exp: experimental values
IF: ionized phenolic compounds
PRE: precipitation
UF: unionized phenolic compounds
